# Defensive Molecules Momilactones A and B: Function, Biosynthesis, Induction and Occurrence

**DOI:** 10.3390/toxins15040241

**Published:** 2023-03-25

**Authors:** Hisashi Kato-Noguchi

**Affiliations:** Department of Applied Biological Science, Faculty of Agriculture, Kagawa University, Miki, Kagawa 761-0795, Japan; kato.hisashi@kagawa-u.ac.jp

**Keywords:** allelopathy, biosynthesis, diterpenoid, *Echinochloa crus-galli*, elicitation, momilactone, *Oryza sativa*, pathogen, rice blast

## Abstract

Labdane-related diterpenoids, momilactones A and B were isolated and identified in rice husks in 1973 and later found in rice leaves, straws, roots, root exudate, other several Poaceae species and the moss species *Calohypnum plumiforme.* The functions of momilactones in rice are well documented. Momilactones in rice plants suppressed the growth of fungal pathogens, indicating the defense function against pathogen attacks. Rice plants also inhibited the growth of adjacent competitive plants through the root secretion of momilactones into their rhizosphere due to the potent growth-inhibitory activity of momilactones, indicating a function in allelopathy. Momilactone-deficient mutants of rice lost their tolerance to pathogens and allelopathic activity, which verifies the involvement of momilactones in both functions. Momilactones also showed pharmacological functions such as anti-leukemia and anti-diabetic activities. Momilactones are synthesized from geranylgeranyl diphosphate through cyclization steps, and the biosynthetic gene cluster is located on chromosome 4 of the rice genome. Pathogen attacks, biotic elicitors such as chitosan and cantharidin, and abiotic elicitors such as UV irradiation and CuCl_2_ elevated momilactone production through jasmonic acid-dependent and independent signaling pathways. Rice allelopathy was also elevated by jasmonic acid, UV irradiation and nutrient deficiency due to nutrient competition with neighboring plants with the increased production and secretion of momilactones. Rice allelopathic activity and the secretion of momilactones into the rice rhizosphere were also induced by either nearby *Echinochloa crus-galli* plants or their root exudates. Certain compounds from *Echinochloa crus-galli* may stimulate the production and secretion of momilactones. This article focuses on the functions, biosynthesis and induction of momilactones and their occurrence in plant species.

## 1. Introduction

**Figure 1:**
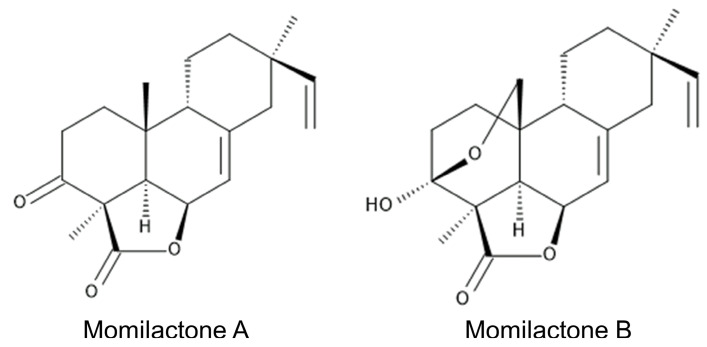
Momilactones.

Labdane-related diterpenoids, momilactones A and B (Figure 1) were first isolated and identified in rice husks as potent germination and growth-inhibitory substances in 1973 [1]. Momilactones were later isolated from rice leaves as phytoalexins against fungal pathogens such as the rice blast fungus *Magnaporthe oryzae* [2,3]. The concentrations of momilactones increased 2 days after infection with *Magnaporthe oryzae*, and momilactones suppressed the further growth of the fungus [4,5]. The fungal elicitors chitosan and cholic acid also induced the accumulation of momilactone A in rice leaves and suspension-cultured rice cells [6,7].

The function of momilactones, especially momilactone A, as phytoalexins has been extensively studied, and the evidence suggests that momilactones may play a role in the rice defense function against fungal pathogens [8,9,10].

The first finding of rice allelopathy was made in field examinations in Arkansas, U.S.A., where 191 of over 5000 rice accessions suppressed the growth of the aquatic weed *Heteranthera limosa* [11]. Allelopathy is defined as the chemical interactions among various plant species [12]. Certain plants release some secondary metabolites, termed allelochemicals, into their immediate environment, and these allelochemicals affect the growth and development of other plant species nearby [13,14,15,16,17]. The observation of rice allelopathy led to large field screening programs. Among over 16,000 rice germplasm collections of the USDA-ARS from 99 countries, 412 rice accessions suppressed the growth of *Heteranthera limosa*, and 145 rice accessions suppressed the growth of *Ammannia coccinea* [18,19]. More than 40 rice cultivars among 1000 rice collections inhibited the growth of *Echinochloa crus-galli* and *Cyperus difformis* [20]. Screening programs in the field and/or laboratories have also been carried out in several other countries, and it was found that certain rice cultivars released allelochemicals from their root systems into their immediate environments, such as rhizosphere soil, cultural solutions and other incubation media [21,22,23,24]. Thereafter, momilactones A and B were again isolated and identified in rice root exudates as rice allelochemicals [25,26]. It was also found that rice plants released momilactones throughout their life cycles with sufficient amounts of momilactones for allelopathy [27,28].

Momilactones are synthesized in rice plants from geranylgeranyl diphosphate, which is also a precursor of other phytoalexins and a plant hormone, gibberellic acid [29]. Momilactones are synthesized and accumulated in rice leaves as phytoalexins and secreted into their root zones as allelochemicals [30,31]. A gene cluster related to momilactone synthesis was found on chromosome 4 of the rice genome. Momilactones were later found in some other Gramineae plant species and the moss species *Calohypnum plumiforme* (syn. *Hypnum plumaeform*) as allelochemicals [32,33,34,35]. This review provides an overview of the functions, biosynthesis, induction and occurrence of momilactones in plant species and highlights the importance of momilactones.

## 2. Defense Function against Pathogens, Microbes and Insects

### 2.1. Rice Blast Fungal Pathogen

Infection with the rice blast pathogen *Magnaporthe oryzae* (syn. *Pyricularia oryzae*; renamed from *Magnaporthe grisea*) induced momilactone A accumulation in rice leaves. The accumulation was abundant at the edges of necrotic lesions, which are symptoms of the infection of leaves [36]. Blast fungus susceptibility diffed among rice cultivars, and tolerance to the fungus correlated positively with momilactone A accumulation in rice leaves [37]. Blast-fungus-resistant rice mutants accumulated momilactone A 2 days after fungus inoculation, and the concentration of momilactone A was 100–400-fold greater than that in wild-type rice and suppressed the further growth of the fungus [4,5]. Exogenously applied momilactone A also suppressed the growth of the fungus on agar media [5]. In addition, the susceptibility of momilactone-deficient rice mutants to the blast fungus was high compared to wild-type rice [38]. These observations suggest that momilactone A may prevent the subsequent spread of the fungus infection through the increased production of momilactone A after pathogen infection.

### 2.2. Other Fungal Pathogens

Momilactones A and B inhibited the growth of the pathogenic fungi *Rhizoctonia solani*, *Blumeria graminis*, *Fusarium oxysporum*, *Fusarium solani*, *Botrytis cinereal* and the *Colletrichum gloesporides* complex [39,40]. Infection with *Xanthomonas oryzae* pv. *oryzae*, which causes bacterial blight, increased jasmonic acid and momilactone A concentrations in rice leaves [41]. Jasmonic acid is a plant defense signaling hormone and induces several defense responses for protection [42,43,44].

### 2.3. Anti-Microbe Activity

Momilactone A inhibited the mycelia growth of the mushroom *Coprinus cinereus* [45] and the cyanobacteria *Microcystis aeruginosa* [46]. Momilactones A and B inhibited the growth of the bacteria *Escherichia coli*, *Pseudomonas putida* (former name, *Pseudomonas ovalis*), *Bacillus cereus* and *Bacillus pumilus* [39].

### 2.4. Insect Attack

An insect attack by the white-back planthopper (*Sogatella furcifera*) induced the accumulation of momilactone A in rice leaves through a jasmonic acid-mediated pathway [47]. The jasmonic acid-mediated pathway is described in Section 7. The digestive waste of the rice brown planthopper (*Nilaparvata lugens*) induced momilactone A and B accumulation in rice leaves. Filtration and heat treatments of digestive wastes reduced their accumulation. A symbiont of the insect, *Serratia marcescens*, in the digestive waste also induced the accumulation of momilactones A and B [48]. The function of momilactones A and B against insect attacks is not clear.

## 3. Function in Allelopathy

A considerable number of rice accessions or cultivars have been found to suppress the growth of several other plant species, including weed species, when these rice and other plants were grown together under field and/or laboratory conditions [11,21,22,23,24,49]. These observations suggest that rice is allelopathic and contains allelochemicals. A compound causing the growth-inhibitory effect of rice was later isolated from its root exudates and identified as momilactone B [25]. Momilactone A was also identified in rice secretory fluid [26]. These investigations suggest that momilactones A and B may function as rice allelochemicals.

### 3.1. Activities of Momilactones A and B as Allelochemicals

Momilactones A and B inhibited the growth of several plant species, including weed species such as *Echinochloa crus-galli* and *Echinochloa colonum*. Both *Echinochloa* species are known as the most noxious weeds in rice fields because of their potential to significantly disturb rice production [50,51]. Momilactones A and B inhibited the root and shoot growth of *Echinochloa crus-galli* at concentrations greater than 3 μM and 1 μM, respectively, and the root and shoot growth of *Echinochloa colonum* at concentrations greater than 10 μM and 1 μM, respectively [52]. Table 1 shows the concentrations of momilactones A and B required for 50% growth inhibition (defined as *IC_50_*) of target plant species. Smaller values of *IC_50_* indicate the higher susceptibly of the target plants to momilactones. On the basis of *IC_50_* values, monocotyledonous weed plant species (*Echinochloa crus-galli*, *Echinochloa colonum*, *Phleum pretense*, *Digitaria sanguinalis* and *Lolium multiflorum*) showed higher susceptibly compared to dicotyledonous plant species (*Arabidopsis thaliana*, *Lepidium sativum*, *Lactuca sativa* and *Medicago sativa*) [52,53,54,55]. In addition, momilactone B showed much higher growth-inhibitory activity than momilactone A, which has also been confirmed by other bioassay systems [55,56,57,58,59].

On the other hand, momilactones A and B showed relatively weak inhibitory activity on rice growth compared to *Echinochloa crus-galli*. The rice roots and shoots were suppressed by momilactones A and B at concentrations greater than 300 μM and 100 μM, respectively [52,53]. Thus, the effect of momilactones on rice was only 1% of that on *Echinochloa crus-galli*, which was inhibited at concentrations greater than 3 μM and 1 μM for roots and shoots, respectively, as described above [52,53]. In addition, momilactones A and B did not cause any visible damage to rice plants at concentrations that were phytotoxic to other plant species [52,53,54,55]. These observations suggest that the toxicity of momilactones A and B to rice plants is much less than that to other plant species. The resistance mechanism of rice to momilactones is unknown. This tolerance may possibly involve either rapid secretion, the insensitivity of the molecular target and/or the degradation of momilactones.

### 3.2. Concentration and Secretion of Momilactones

The endogenous concentrations of momilactones A and B, respectively, in rice were 4.5 μg/g and 3.0 μg/g of rice straw [60] and 4.9 μg/g and 2.9 μg/g of rice husks [61]. Momilactone B was found in rice seedlings 7 days after germination, and the concentrations of momilactones A and B increased until day 80 after germination, which is when flowering is initiated [52,62,63,64]. The 80-day-old rice plants contained momilactones A and B at 140 μg/g and 95 μg/g in rice plants, respectively [52,64]. Considering their reported concentrations, the ratio of momilactone A to momilactone B is 1.5–1.6.

The secretion of momilactone B from rice roots was observed 3 days after germination [62]. The levels of momilactone A and B secretion increased up to day 80 after germination and decreased thereafter [52,63]. The secretion levels of momilactones A and B at day 80 were 1.1 and 2.3 μg per plant per day, respectively [52,63], which indicates that the secretion ratio of momilactone B to momilactone A is 2.1. The observation suggests that rice secretes momilactones A and B into its rhizosphere throughout its entire life cycle, and the secretion increases until flowering initiation. Thus, it may be possible that rice allelopathy increases over this time frame. In addition, momilactone B was secreted at a higher rate than momilactone A, even though the concentration of momilactone A is higher than that of momilactone B in rice plants, which suggests that momilactone B may be preferentially secreted into the rhizosphere over momilactone A. Plants are reported to secrete a wide range of compounds from their roots through their cell membranes, for example, by proton-pumping mechanisms, plasmalemma-derived exudation and endoplasmic-derived exudation [65,66,67]. However, the mechanism of the exudation of momilactones from rice roots is unknown.

### 3.3. Contribution of Momilactones to Rice Allelopathy

When eight cultivars of rice seedlings (7 days old) were incubated for four days with *Echinochloa crus-galli* seedlings (4 days old) in a buffered bioassay medium, all rice cultivars suppressed the growth of *Echinochloa crus-galli* with different suppression levels. All rice cultivars produced and secreted momilactones A and B into the media, and the concentrations of momilactones A and B in the media were 0.21–1.45 μM and 0.66–3.84 μM, respectively [53]. Based on the growth-inhibitory activity and secreted amounts of momilactones A and B in the media, momilactone A may only account for 1.0–4.9% of the observed growth inhibition of *Echinochloa crus-galli* by the respective rice cultivars. By contrast, momilactone B may account for 58.8–81.9% of the observed growth inhibition. In addition, the momilactone B concentration in the media was significantly (*p* < 0.01) correlated with the extent of the growth suppression of *Echinochloa crus-galli* by these eight rice cultivars [53]. A similar correlation was also found between the level of momilactone B secretion and the extent of the growth suppression of *Lactuca sativa* by these rice cultivars [68,69]. The observations suggest that momilactone B may be a major contributor to the allelopathic activity of rice, and the secretion levels of momilactone B reflect the variation in allelopathic activity observed rice cultivars. The leaf, straw and husk extracts of 41 rice cultivars differed in their growth-inhibitory activity against *Alisma plantago-aquatica*. The concentration of momilactone B in the extracts was also correlated with the inhibitory activity of the extracts [70].

### 3.4. Genetic Evidence for Momilactones in Rice Allelopathy

Momilactone-biosynthesis-deficient mutants (*cps4* and *ksl4*) were obtained through insertion gene knockouts for OsCPS4 and OsKSL4 [71,72], which is described in Section 5. Allelopathic activity after removing all *syn*-copalyl diphosphate-derived labdane-related diterpenoids (*cps4* mutant) or, more selectively, only momilactones (*ksl4* mutant) was compared to the respective wild-type rice. The wild types showed allelopathic activity, whereas both mutants lost this activity [73]. The investigation suggests that the loss of allelopathic activity may be attributed to the specific loss of momilactones, which verifies the involvement of momilactones in rice allelopathy.

### 3.5. Inhibitory Mechanism

Molecular targets of momilactone B were investigated through SDS-PAGE and two-dimensional gel electrophoresis with MALDI-TOF-MS. Momilactone B suppressed the germination of *Arabidopsis thaliana* and inhibited the breakdown of the storage proteins cruciferina, cruciferin 2 and cruciferin 3 during germination [74]. The breakdown of these proteins is essential to construct cell structures for germination and seedling growth [75,76,77]. The application of momilactone B to *Arabidopsis thaliana* seedlings inhibited the accumulation of amyrin synthase LUP2, subtilisin-like serine protease, β-glucosidase and malate synthase [78]. Those proteins are involved in the production of intermediates and metabolic turnover for cell structures [79,80,81,82]. On the contrary, momilactone B induced the accumulation of translationally controlled tumor protein, 1-cysteine peroxiredoxin 1 and glutathione-*S*-transferase [75]. These proteins elevate the tolerance to drought and oxidative stress conditions [83,84,85]. In addition, glutathione-*S*-transferase showed herbicide detoxification activity [86], and 1-cysteine peroxiredoxin 1 showed germination-inhibitory activity under unfavorable conditions [87]. These observations suggest that momilactone B may cause growth inhibition through the suppression of metabolic turnover and the production of intermediates and induce tolerance to stress conditions.

### 3.6. Induction of Rice Allelopathy and Momilactone

The allelopathic activity of rice was increased by nutrient deficiency, which is often caused by competition with neighboring plants [88,89,90]. The nutrient-deficient condition also increased the production and secretion of momilactone B from rice [91]. In addition, the allelopathic activity of rice was also elevated by either nearby *Echinochloa crus-galli* plants or their root exudates [91,92,93,94]. This elevation was not only owing to nutrient competition between rice and *Echinochloa crus-galli* [95,96]. The momilactone B concentration in rice and its secretion level from rice were also increased by either *Echinochloa crus-galli* or its root exudates. Rice may recognize certain components of the root exudation of *Echinochloa crus-galli*, and the compounds trigger the increased production and secretion of momilactone B [91,95,96]. Other weed species, namely, *Eclipta prostate* and *Leptochola chinensis*, also increased the secretion of momilactone B [97].

Rice allelopathic activity was also elevated by jasmonic acid [98]. The application of jasmonic acid and cantharidin with UV irradiation also increased the concentration of momilactone B in rice and the secretion levels of momilactones from rice roots into its rhizosphere [99]. As momilactones, especially momilactone B, have strong allelopathic activity, as described previously, such increasing secretion levels of momilactones may provide a competitive advantage for rice through the suppression of the growth of nearby competing plant species.

## 4. Pharmacological Activity

### 4.1. Anticancer Activity

Momilactones A and B showed growth suppression activity in the murine leukemia P399 cell line [100]. Momilactones A and B induced apoptosis in acute promyelocytic leukemia HL-60 and multiple myeloma U266 cell lines through the activation of apoptosis-inducing factors such as caspase-3 [101]. Momilactone B also induced G_1_ arrest in the cell cycle and apoptosis in the human leukemia U937 cell line through the suppression of pRB phosphorylation and the induction of the kinase inhibitor p21 [102], and it induced apoptosis in human leukemia T cells through the activation of caspase [103] and in human breast cancer cells through signal transducer and activator of transcription 5 and a caspase-3-dependent pathway [104]. Momilactone B showed cytotoxic activity in the human colon cancer HT-29 and SW620 cell lines [105].

### 4.2. Anti-Inflammatory Activity

Momilactone A suppressed the inflammatory response in mouse macrophage RAW264.7 cells through a reduction in NO production and iNOS mRNA expression [106].

### 4.3. Anti-Diabetic Activity

Momilactones A and B suppressed pancreatic α-amylase, α-glucosidase and trypsin activity in vitro, which indicates that momilactones A and B may work as diabetes inhibitors [107,108].

### 4.4. Anti-Ketosis Activity

Momilactone B inhibited ketosis in vitro through the suppression of the mitochondrial enzyme 3-hydroxy-3-methylglutaryl-CoA synthase-2, which converts acetyl-CoA to ketone bodies [109].

### 4.5. Anti-Melanogenic Activity

Momilactone B inhibited the accumulation of melanin in B16 melanocytes through the suppression of protein kinase A signaling and tyrosinase-related proteins [110].

## 5. Biosynthesis and Related Genes

**Figure 2:**
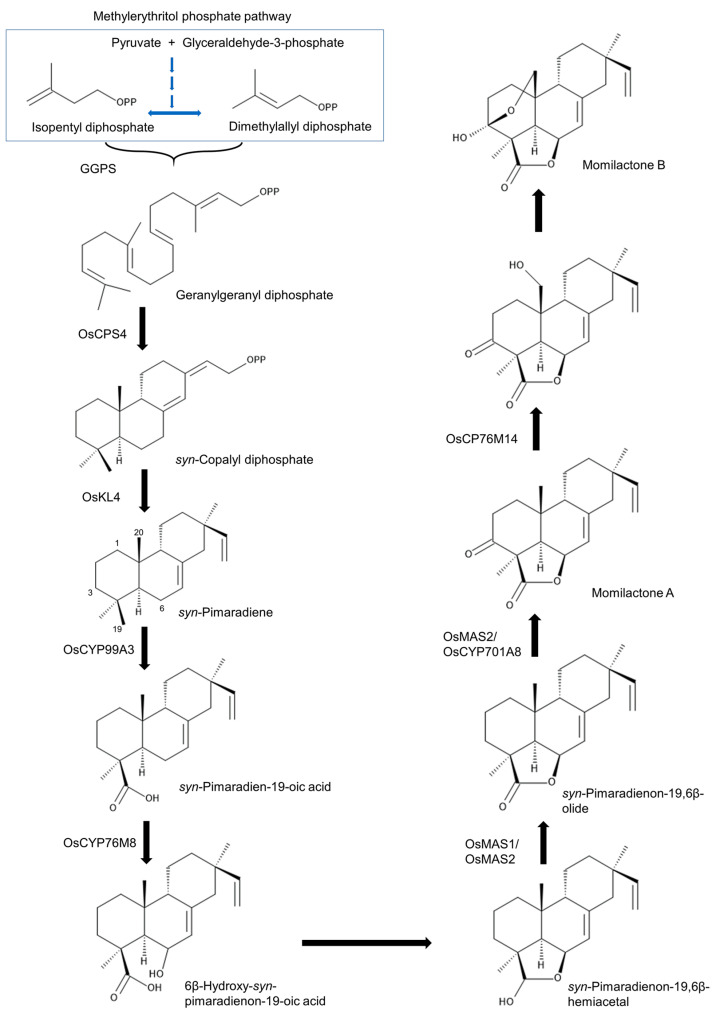
Biosynthetic pathway of momilactones in rice.

Geranylgeranyl diphosphate (GGDP) is the precursor of the plant hormone gibberellin and rice diterpenoid phytoalexins such as oryzalexins and phytocassanes, including momilactones [29]. GGDP is synthesized by GGDP synthase (GGPS) from two five-carbon isoprenoids, isopentyl diphosphate or dimethylallyl diphosphate, which are synthesized through the methylerythritol phosphate pathway from pyruvate and glyceraldehyde-3-phosphate [111] (Figure 2).

GGDP is cyclized into *syn*-copalyl diphosphate (*syn*-CDP) by CDP synthases (OsCPS4). *syn*-CDP is further cyclized into *syn*-pimaradiene by *ent*-kaurene synthase-like 4 (OsKSL4) [112,113,114]. cDNA encoding OsCPS4 was obtained from UV-irradiated rice leaves [115]. *OsCOS4* and *OsKSL4* are located close to each other on chromosome 4 and were demonstrated to have sequential activity producing *syn*-CDP and *syn*-pimaradiene [116,117].

**Figure 3:**
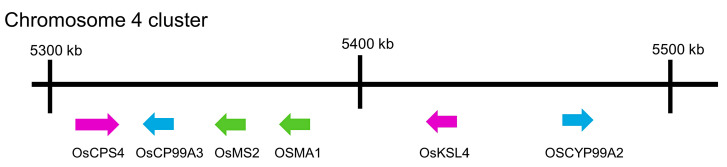
Gene cluster on chromosome 4 of rice genome for momilactone biosynthesis.

Cytochrome P450 enzymes (CYPs) are involved in the further metabolism of *syn*-pimaradiene. OsCYP99A3 oxidizes the C19 methyl of *syn*-pimaradiene into *syn*-pimaradien-19-oic acid [118,119], and OsCPY76M8 then hydroxylates its C6 position into 6β-hydroxy-*syn*-pimaradienon-19-oic acid, followed by the spontaneous closure of the ring between C19 and C6, which forms *syn*-pimaradienon-19,6β-hemiacetal [120]. Momilactone synthase (OsMS1 or OsMS2) converts the C19 hydroxyl group into a ketone to form *syn*-pimaradienon-19,6β-olide [120]. OsMS2 (or OsCPY701A8) then catalyzes C3 hydroxy into a ketone, forming momilactone A [120]. C20 hydroxylation of momilactone A by OsCP76M14 leads to the spontaneous closure of the hemiacetal ring and forms momilactone B [120,121]. The momilactone-synthesis-related genes *OsCPS4*, *OsKSL4*, *CYP99A2*, *CYP99A3*, *OsMS1* and *OsMS2* were reported to be located on chromosome 4 in plastids of rice cells [32,122] (Figure 3), which indicates that momilactones may play an important ecological role in rice evolution because of the presence of a dedicated biosynthetic gene cluster in the rice genome.

## 6. Momilactone Induction

Plants often respond by increasing their production of certain phytoalexins when they are attacked by pathogens and insects. The reaction involves the induction of active oxygen species, lignification, protease inhibitors and some enzymes, such as chitinase and β-glucanase. Plant defense reactions are also induced by a variety of biological, chemical and physical elicitors, such as oligosaccharides, cantharidin and UV irradiation [123,124,125]. Momilactone A and B production and accumulation were also induced by these elicitors.

### 6.1. Biotic Elicitors

Chitosan (oligosaccharide) is a deacetylated derivative of chitin, which is a long-chain polymer of *N*-acetylglucosamine and a primary component of fungal cell walls, arthropod exoskeletons and insect exuviae [126,127]. Chitosan increased the accumulation of momilactone A in rice leaves and suspension-cultured rice cells [6,7] and increased the tolerance of rice to the rice blight pathogen *Fusarium oxysporum* [128]. *N*-Acetylchitooligosaccharides, which are released from the cell walls of pathogenic fungi, also induced the accumulation of momilactones A and B in suspension-cultured rice cells, and their accumulation was 100–500 g/g of cultured cells, which is a sufficient concentration to prevent the growth of pathogenic fungi [129].

Tetraglucosyl glucitol [β-(1,3/1,6)-derived glucan] increased momilactone A production in rice cells [130]. Cantharidin, a protein serine/threonine phosphatase inhibitor contained in some insects, has been shown to mimic elicitor action in plants and to activate defense responses [131,132], and it increased the concentrations of momilactones A and B in rice [132,133] and the secretion level of momilactone B [134]. Cerebrosides (monoglycosylceramides), which are important components of animal cell membranes, induced β-glucanase, chitinase and peroxidase-encoding transcripts and enhanced the production of momilactone A [36,135]. The application of methionine also increased the momilactone A concentration in rice leaves. A free radical scavenger, Tiron (disodium 4,5-dihydroxy-1,3-benzenedisulfonate), increased the momilactone A concentration, which suggests that active oxygen species may stimulate methionine-induced momilactone A production [136].

### 6.2. Abiotic Elicitors

UV irradiation (254 nm, 20 min) increased momilactone A and B concentrations in rice leaves, and the maximum accumulation was found 3 days after UV irradiation [3,137]. The increase in the levels of momilactone A differed among rice varieties, and blast-resistant rice varieties accumulated more momilactone A than susceptible rice varieties [37].

The application of CuCl_2_ to rice leaves also induced momilactone A accumulation. The accumulation was detected 12 h after application and reached maximum accumulation at 72 h. FeCl_2_ and HgCl_2_ also increased momilactone A accumulation by 37% and 20% compared to CuCl_2_ application, respectively [138]. The application of CuCl_2_ to rice leaves induced jasmonic acid and momilactone A. Jasmonic acid biosynthesis inhibitors, quinacrine, nordihydroguaiaretic acid and salicylhydroxamic acid, suppressed momilactone A accumulation after the application of CuCl_2_. However, additional jasmonic acid application induced momilactone A accumulation after the application of CuCl_2_ and jasmonic acid biosynthesis inhibitors [139]. These observations suggest that CuCl_2_ increased the concentration of jasmonic acid in the leaves, and jasmonic acid then stimulated the biosynthesis of momilactone A. In addition, the application of CuCl_2_ and FeCl_2_ increased the production and secretion levels of momilactone B in rice and its allelopathic activity [133].

Other metal ions, such as silver, potassium, calcium, sodium zinc and magnesium, also increased the accumulation of momilactone A in suspension-cultured rice cells [140]. The air pollutant sulfur dioxide (SO_2_) induced reddish-brown necrotic spots on rice leaves and increased the momilactone A concentration in the leaves [141]. A fungicide, 2,2-dichloro-3,3-dimethyl cyclopropane carboxylic acid, also induced the accumulation of momilactones A and B in rice leaves [2]. Protein synthesis inhibitor herbicides, pretilachlor and butachlor, increased momilactone A accumulation in rice leaves [142].

## 7. Induction Signaling

**Figure 4:**
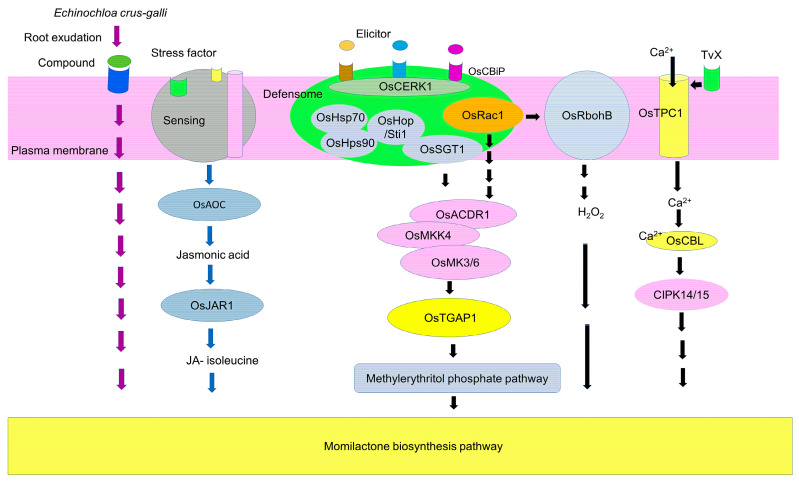
Signaling pathway for the elicitation of rice momilactone biosynthesis.

The generation of elicitor fragments after pathogen and insect attacks may occur through the induction of chitinase and β-1,3-glucanase [143,144]. Elicitor fragments such as *N*-acetylchitooligosaccharide induced the formation of hetero-oligomer complexes of OsCEBiP (chitin elicitor binding protein) and OsCERK1 (chitin elicitor receptor kinase) [145]. OsCERK1 is part of the defensome complex at the plasma membrane (Figure 4). The defensome contains OsHsp70 (heat shock protein 70), OsHps90, OsHop/Sti1 (Hsp70/Hsp90 organizing protein/stress-induced protein 1), OsSGT1 (suppressor of G/two allele of *Skp*1) and OsRAR1 (required for *Mla12* resistance) as molecular chaperone proteins and co-chaperon-like proteins [146,147,148]. OsRac1 (small-specific Rho-type GTPase), which is another important component, may cause mitogen-activated protein kinase (MAPK) signal cascades [149]. The earliest MAPK signaling step is OsACDR1 (accelerated cell death and resistance 1), followed by OsMKK4 and then OsMK3 and/or OsMK6 [150,151]. OsTGAP1 (TGA factor for phytoalexin production 1) may then induce the methylerythritol phosphate pathway and the expression of momilactone biosynthetic genes, including OsKSL4 [152,153].

OsRac1 may interact with OsRbohB (respiratory burst oxidase homolog B) in a Ca^2+^-dependent manner [154]. The constitutive expression of OsRac1 causes an increase in H_2_O_2_ production, *OsCP2* transcripts and momilactone A accumulation in rice [155].

*Tricoderma viride*-derived xylanase (TvX) requires specific receptors [156] and increased cytosolic Ca^2+^ within minutes [157,158]. Cytosolic Ca^2+^ induction by TvX is partly mediated by the plasma membrane putative voltage-gated cation channel OsTPC1 [158]. TvX-induced signaling targets Ca^2+^-sensing calcineurin B-like proteins (OsCBL) and CBL-interacting protein kinases (CIPK14 and 15), which may act as Ca^2+^ sensors [157]. Increased momilactone production was found 24 h after TvX application [157,158].

Exogenous jasmonic acid (JA) induced the accumulation of momilactones [159]. Jasmonic acid production in plants after exposure to stress factors is initiated by the peroxidation of linolenic acid, followed by allene oxide cyclase-mediated epoxide formation, cyclization by allene oxide cyclase (OsAOC) and β-oxidation [159,160]. The produced jasmonic acid is then conjugated with isoleucine by OsJAR1 (Jasmonate Resistant 1), resulting in the formation of JA-isoleucine (JA-Il). JA-Il may then stimulate momilactone synthesis [159]. The exogenous application of salicylic acid also induced momilactone accumulation in rice. However, the mechanism of salicylic acid induction of momilactones remains unclear [161].

## 8. Occurrence of Momilactone

Momilactones A and B were first isolated from seed husks of *Oryza sativa* cv. Koshihikari [1] and then found in the leaves of *Oryza sativa* [2], but these studies did not clearly mention the cultivar or accession of the rice. Momilactones A and B were found in whole plants of rice, including their roots [60,64], and in multiple rice cultivars [53,68,69,70,162]. The concentrations of momilactones A and B were determined in the leaves of 69 rice cultivars from World Rice Core Collections, and in 64 and 31 cultivars, the presence of momilactones A and B were detected, respectively. The concentrations of momilactones A and B varied among these cultivars. The maximum amount of momilactone A was recorded in the cultivar Urasan at 495 nmol/g leaf, but the exact value for momilactone B was not reported. The concentrations of momilactones A and B in the leaves were greater in Japonica-type cultivars than in Indica-type cultivars [162].

Wild rice species such as *Oryza rufipogon*, *O. burthii*, *O. glaberrima*, *O. glumaepatula*, *O. meridionalis*, *O. punctatas* and *O. brachyatha* also contained momilactones A and B. The concentrations of momilactone A were 0.97–667 nmol/g leaf [162]. Momilactone biosynthesis genes of *O. punctatas* (*OpCPS4*, *OpCYP99A*, *OpMS1*, *OpMS2*, *OpKSL4* and *OpCYP99A*) form a gene cluster on the same chromosome [163]. These genes are equivalent to rice *OsCPS4*, *OsCYP99A*, *OsMS1*, *OsMS2*, *OsKSL4* and *OsCYP99A* genes, respectively. A gene cluster for momilactone biosynthesis was also found in *Echinochloa crus-galli*. The gene cluster contains only single copies of *EcCY99A* and *EcMS*, and its gene sequence on the chromosome is different from that in rice [164]. However, the endogenous concentration of momilactones in *Echinochloa crus-galli* has not yet been reported.

Momilactones A and B were also found in the moss species *Calohypnum plumiforme* (syn. *Hypnum plumaeform*), which is quite taxonomically distinct from rice [33,34,35]. *Calohypnum plumiforme* belongs to the Hypnaceae family of the Bryophyta division, often dominates in plant communities and forms large pure colonies in sunny places in lowland to upland areas, including marshy places in eastern Asia [165,166]. Momilactones are also synthesized from GGDP in the moss. GGDP is cyclized to *syn*-pimaradiene by diterpene cyclase (CpDTC1/HpDTC1) [167]. *syn*-Pimaradiene is catalyzed into 3β-hydroxy-*syn*-pimaradienon-19,6β-olide by CpCYP770A14 and CpCYP964A1. 3β-Hydroxy-*syn*-pimaradienon-19,6β-olide is then metabolized to momilactone A by momilactone synthase (CpMS) [168]. Those genes also form a gene cluster in the order *CpMS*, *CpCYP970A14*, *CpDTC1*/*HpDTC1* and *CpCYP964A1* on the same chromosome [168].

Momilactone A and B concentrations in the moss were 58.7 μg/g and 23.4 μg/g dry weight of the moss, respectively. The moss also secretes momilactones A and B into the rhizosphere at ratios of 4.0 μg/g and 6.3 μg/g dry weight of the moss, respectively, which were 7.3% and 27% of the endogenous concentrations of momilactones A and B in the moss [35]. The observations suggest that the moss selectively secretes momilactone B into the rhizosphere rather than momilactone A. UV irradiation, jasmonic acid and cantharidin also increased the production and secretion levels of momilactones A and B [169]. These observations suggest that elicitors and/or pathogen attacks may increase the production and secretion levels of momilactones A and B in the moss. Momilactones A and B secreted from moss are also able to suppress the growth of neighboring plant species. Therefore, momilactones in the moss may function in the defense against pathogen attacks and allelopathy.

## 9. Conclusions

The literature reviewed here demonstrates an important role for momilactones in the defense function and allelopathic function. Momilactones in rice plants may provide resistance to fungal pathogen attacks, and momilactones in rice root exudate may provide rice with the ability to compete with neighboring plant species, which was confirmed with momilactone-deficient mutants. The momilactone biosynthesis pathway and related genes have been investigated by many researchers. The elicitation of momilactone production and secretion and the endogenous signaling cascades involved in the elicitation are also well documented. These findings suggest that the allelopathic and defense functions of momilactones may play important ecological roles in rice evolution because of the existence of a dedicated biosynthetic gene cluster in the genome. However, the mechanism and molecular targets of momilactone functions remain unknown. Momilactones A and B did not cause growth suppression or any visible damage to rice plants at concentrations that were phytotoxic to other plant species. The resistance mechanism of rice to momilactones is also unknown. It is worth investigating the mechanism underlying this tolerance for developing resistant crop plants. The potential of momilactones to serve as endogenous natural fungicides and herbicides provides significant benefits when applied to other important crops. The identification of momilactones may provide a molecular marker for breeding and engineering directed at increasing defense and allelopathic abilities. In addition, momilactones have shown anti-leukemia and anti-diabetic activities. Further investigations are necessary to develop their medical applications.

## Figures and Tables

**Table 1:** The concentrations (μM) required for 50% growth inhibition (*IC_50_*) of various plant species.

	Momilactone A		Momilactone B		Reference
Target Plant Species	Roots	Shoots	Roots	Shoots	
*Echinochola crus-gall*	28.7	46.4	6.1	6.3	[53]
*Echinochloa colonum*	65.4	240	5.04	12.5	[52]
*Phleum pratense*	76.5	157	5.6	7.9	[55]
*Digitaria sanguinalis*	98.5	275	9.5	12.4	[55]
*Lolium multiflorum*	91.9	138	6.9	6.5	[55]
*Arabidopsis thiliana*	203	84.4	12	6.5	[54]
*Lepidium sativum*	425	285	6.3	4.6	[35]
*Lactuca sativa*	472	395	54.3	77.9	[55]
*Medicago sativa*	379	315	67.8	82.4	[55]
